# Supplementary material for: The tumor suppressor TMEM127 regulates insulin sensitivity in a tissue-specific manner
Source: Nat Commun. 2019 Oct 17;10:4720. doi: 10.1038/s41467-019-12661-0 (PMC6797792; doi:10.1038/s41467-019-12661-0)
Supplement: Supplementary file 1 — Supplementary Information [file 41467_2019_12661_MOESM1_ESM.pdf]

Supplementary Information

The tumor suppressor TMEM127 regulates insulin sensitivity in a tissue-specific manner

Srikantan et al.

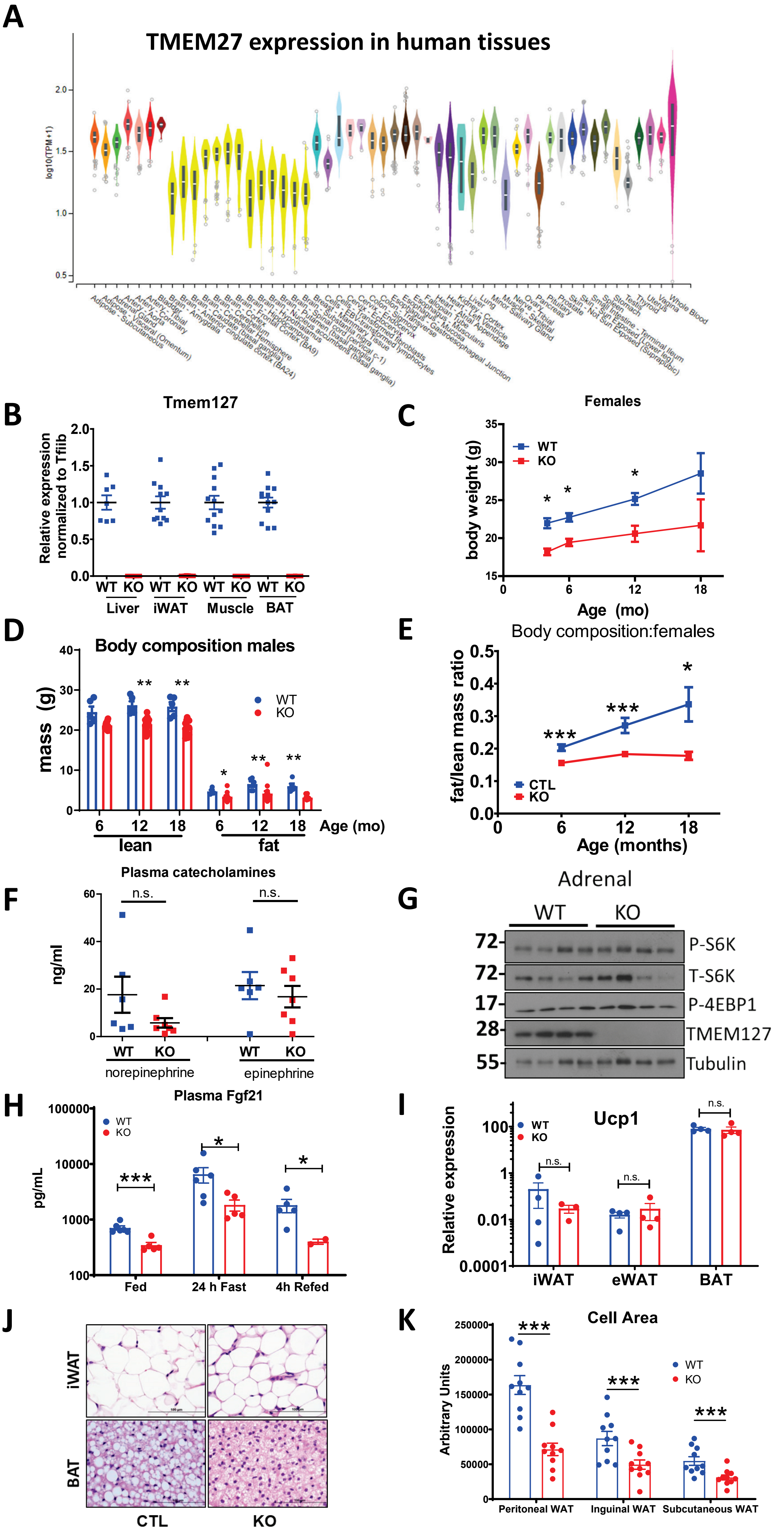
A
TMEM27 expression in human tissues
log10(TPM+1)
B
Tmem127
Relative expression normalized to Tfiib
WT KO WT KO WT KO WT KO
Liver iWAT Muscle BAT
C
Females
WT
KO
body weight (g)
Age (mo)
D
Body composition males
mass (g)
WT
KO
lean
fat
Age (mo)
E
Body composition:females
fat/lean mass ratio
CTL
KO
Age (months)
F
Plasma catecholamines
ng/ml
n.s.
norepinephrine
epinephrine
G
Adrenal
WT
KO
P-S6K
T-S6K
P-4EBP1
TMEM127
Tubulin
H
Plasma Fgf21
pg/mL
Fed
24 h Fast
4h Refed
I
Ucp1
Relative expression
iWAT
eWAT
BAT
J
iWAT
BAT
CTL
KO
K
Cell Area
Arbitrary Units
Peritoneal WAT
Inguinal WAT
Subcutaneous WAT

Supplementary Fig 1. **Global deletion of Tmem127 impairs growth and results in low adiposity** A) TMEM127 expression in different human tissues by RNA seq from the GTEX project (ENSG00000135956.4, data represent Log10 transformed TPM(transcript per million)+1 values; plots show median and 25th and 75th percentiles, points are outliers (above 1.5x the interquartile range), obtained from the GTEX portal, V7 release (including 11,688 samples from 714 donors) on 06/12/2019); B) Relative Tmem127 expression in tissues from WT and Tmem127 KO mice (n=7 and 12 per genotype, respectively), measured by RT-PCR and normalized to the Tfiib gene expression; C) Body weight of female wild-type (WT) and Tmem127 knockout (KO) mice (n=8 and 10 per genotype, respectively) from 6-22 months of age; D) fat and lean mass of male WT and KO mice at the indicated ages (n=5-14 per genotype, respectively); E) fat mass/lean mass ratio of female WT and KO mice across ages (n=8 or 10 per genotype, respectively); F) Plasma levels of norepinephrine and epinephrine of adult male WT (n=6) and KO (n=7) mice measured by gas chromatography/mass spectrometry (GC/MS); G) Western blot of adrenal lysates of WT and KO adult male mice (n=4 per genotype), probed for mTORC1 targets S6K (phosphorylated S6K, pS6K and total S6K) and p-4EBP1; TMEM127 and loading control antibodies; H) Plasma levels of Fgf21 of adult male WT and KO mice under regular fed conditions, after 24 h fasting and after 4h refeeding (n=6 per genotype); I) Relative inguinal (iWAT), epididymal (eWAT) and brown adipose tissue (BAT) mRNA expression of the Ucp1 gene expression in adult WT and KO mice (n=10 or 12 per genotype, respectively) measured by real-time PCR (RT-PCR) and normalized to TfIIb gene; J) Representative HE–stained sections of inguinal WAT of adult male WT and KO mice (scale bar is 100µM); K) Diameter of the designated adipocytes from adult male WT and KO mice (n=6 per genotype). Data were analyzed by Student's t-test. Values are expressed as mean +/- s.e.m. *P<0.05; **P<0.01; ***P<0.001. Adult mice were 9-12months of age. Source data are provided as a Source Data file.

Supplementary Table 1. Pre-birth and neonatal *Tmem127* knockout mouse fitness based on genotype distribution

Age	Total number of litters	Total number of embryos	KOs (n)	Hets (n)	WT (n)	P value
E13.5	44	299	78	137	84	0.66
E18/E19	9	70	21	30	19	0.63
P0	23	154	27	95	32	0.03

E=embryonic day; P=postnatal day; KO=knockout; WT=wild-type; Hets= heterozygotes; p-value: Z-statistic, significance level

Supplementary Table 2. Perinatal (pre- and post-birth) body weight of *Tmem127* knockout mice

	KO			WT			
Age	mean (g)	SEM	n	mean (g)	SEM	n	p value
E18.5	1.00	0.06	8	1.02	0.07	8	0.55
P7	3.28	0.21	10	3.79	0.12	18	0.03
P14	5.22	0.26	10	6.24	0.17	18	0.00
P21	6.60	0.47	10	8.85	0.20	18	< 0.0001
P30	11.48	1.00	9	15.77	0.34	16	< 0.0001
P45	17.00	0.75	7	21.48	0.35	15	< 0.0001
P60	20.19	0.99	5	24.57	0.37	14	< 0.0001

E=embryonic day; P=postnatal day; E=embryonic day; P=postnatal day; KO=knockout; WT=wild-type; Hets= heterozygotes; SEM=standard error of the means; p-value: Student's t-test

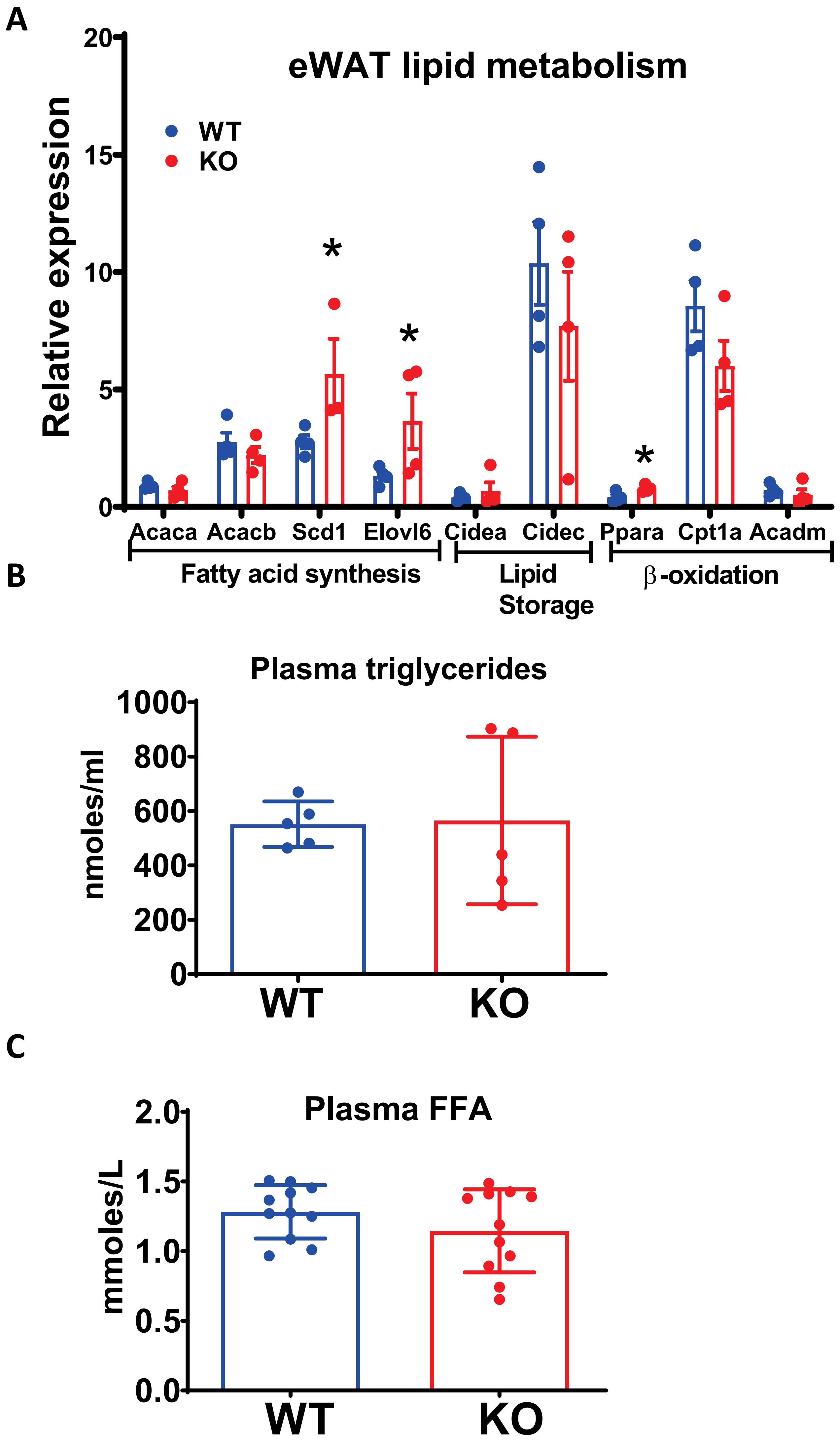
A
eWAT lipid metabolism
Relative expression
WT
KO
0
5
10
15
20
*
*
*
Acaca
Acacb
Scd1
Elovl6
Cidea
Cidec
Ppara
Cpt1a
Acadm
Fatty acid synthesis
Lipid Storage
β-oxidation
B
Plasma triglycerides
nmoles/ml
0
200
400
600
800
1000
WT
KO
C
Plasma FFA
mmoles/L
0.0
0.5
1.0
1.5
2.0
WT
KO

Supplementary Fig 2. **Global Tmem127 deletion impairs hepatic lipogenesis.** A) Relative eWAT mRNA expression of the indicated fatty acid synthesis, storage, oxidation and transport gene expression in adult WT and KO mice (n=10 or 12 per genotype, respectively) measured by real-time PCR (RT-PCR) and normalized to TfIIb gene; B) Plasma triglyceride content in fed adult WT and KO mice (n=5 per genotype); C) Plasma free fatty acids in fed adult WT and KO mice (n=13 per genotype). Data were analyzed by Student's t-test. Values are expressed as mean +/- s.e.m. *P<0.05; **P<0.01; ***P<0.001. Adult mice were 9-12months of age. Source data are provided as a Source Data file.

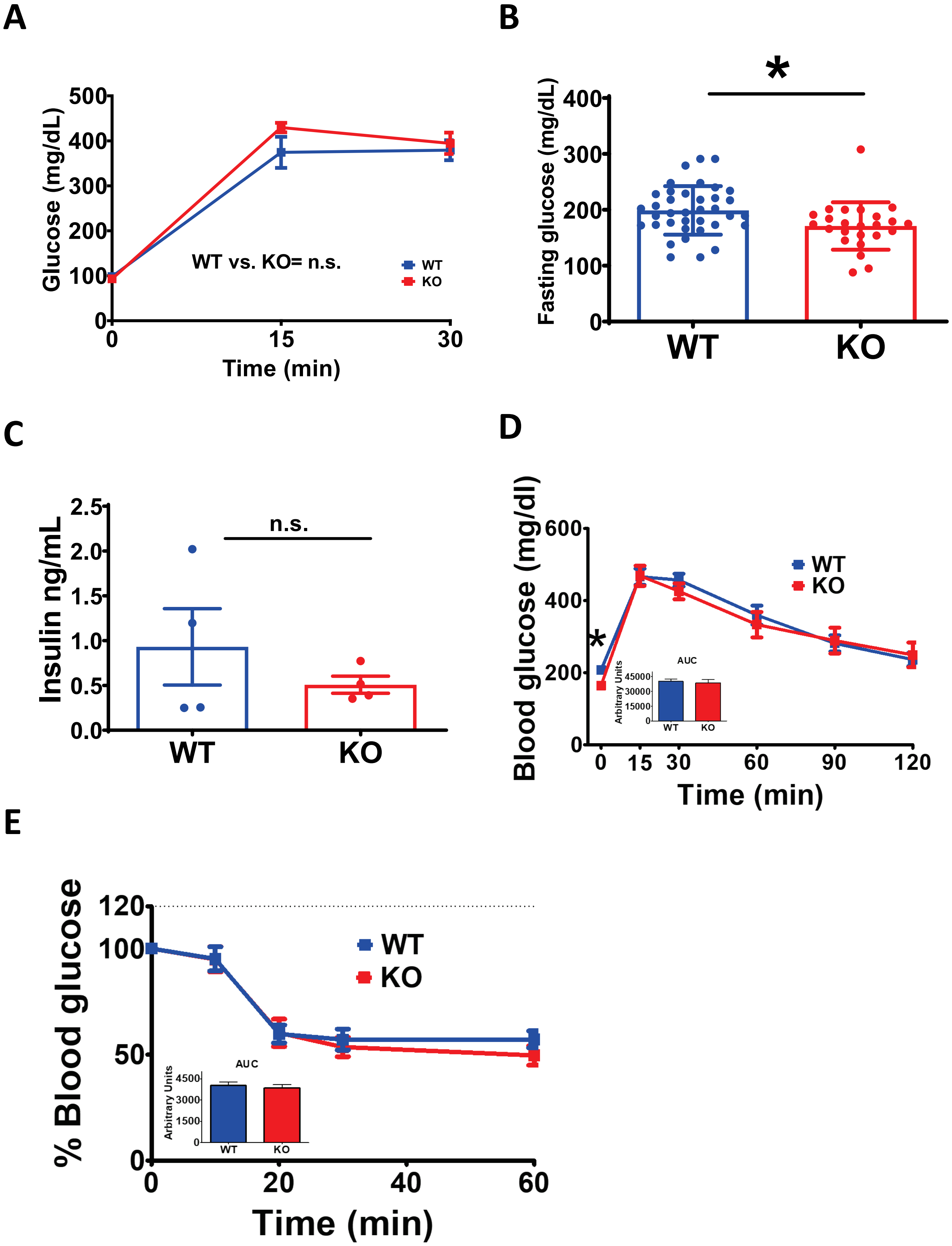
A
Glucose (mg/dL)
500
400
300
200
100
0
0
15
30
Time (min)
WT vs. KO= n.s.
WT
KO
B
*
Fasting glucose (mg/dL)
400
300
200
100
0
WT
KO
C
Insulin ng/mL
2.5
2.0
1.5
1.0
0.5
0.0
n.s.
WT
KO
D
Blood glucose (mg/dl)
600
400
200
0
*
0
15
30
60
90
120
Time (min)
WT
KO
AUC
Arbitrary Units
45000
30000
15000
0
WT
KO
E
% Blood glucose
120
100
50
0
0
20
40
60
Time (min)
WT
KO
AUC
Arbitrary Units
4500
3000
1500
0
WT
KO

Supplementary Fig 3. **Tmem127 deletion leads to hypoinsulinemia and improved insulin tolerance.** A) Blood glucose levels from the GSIS test from Fig 2D-performed in adult male WT and KO mice (n=4 per genotype; B)Fasting (6 hours) blood glucose of 3-6 month-old (young) WT and Tmem127 KO male mice (n=37 and 24 per genotype, respectively); C) Fasting (6 hours) serum insulin of 3-6 month-old (young) WT and Tmem127 KO male mice (n=4 per genotype); D) Glucose tolerance test (GTT) and area under the curve (AUC, inset graph) of 3-6 month-old (young) WT and Tmem127 KO male mice (n=7 and 8 per genotype, respectively); E) Insulin tolerance test (ITT) and AUC (inset) of 3-6 month-old (young) WT and Tmem127 KO male mice (n=7 and 8 per genotype, respectively). Data were analyzed by Student's t-test or two-way ANOVA (in the case of GSIS). Values are expressed as mean +/- s.e.m. *P<0.05; **P<0.01; ***P<0.001. Adult mice were 9-12months of age. Source data are provided as a Source Data file.

A

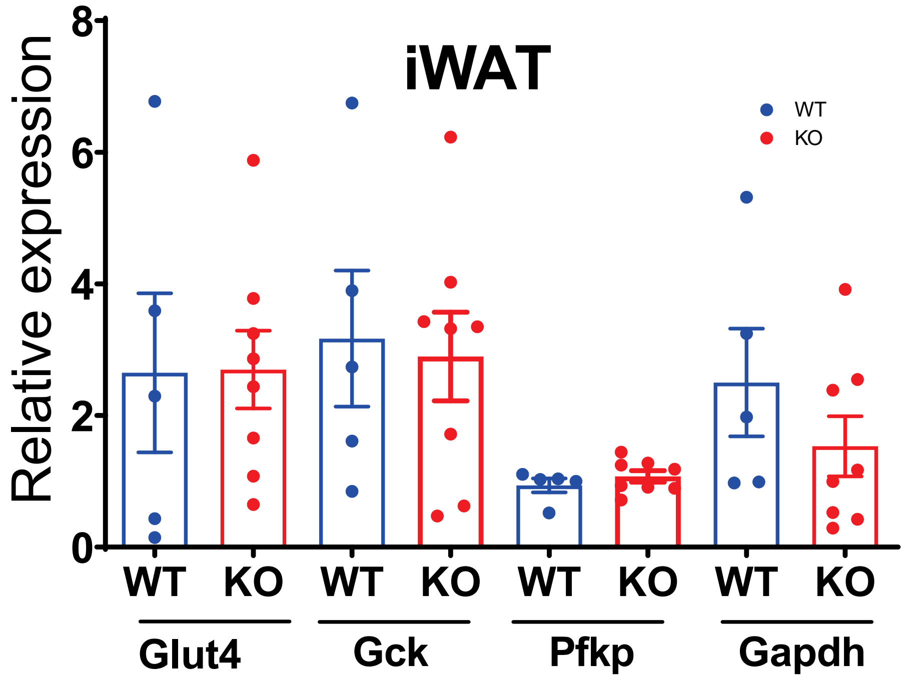
iWAT
WT
KO
Relative expression
0
2
4
6
8
WT
KO
WT
KO
WT
KO
WT
KO
Glut4
Gck
Pfkp
Gapdh

B

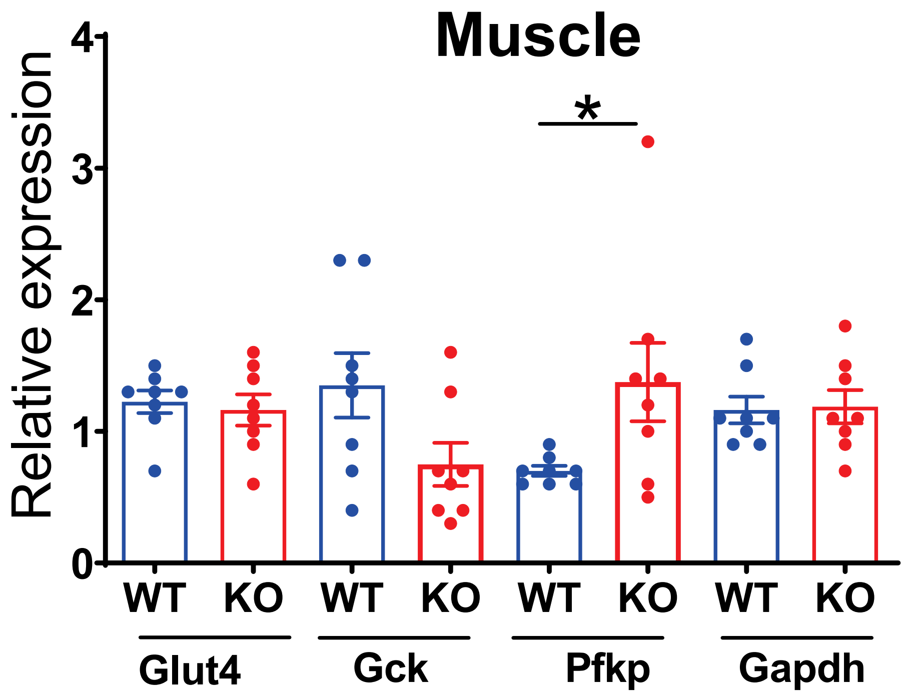
Muscle
*
Relative expression
0
1
2
3
4
WT
KO
WT
KO
WT
KO
WT
KO
Glut4
Gck
Pfkp
Gapdh

Supplementary Fig 4. **Tmem127 deletion decreases hepatic gluconeogenesis and increases insulin signaling.** A) Relative iWAT mRNA expression of the indicated glucose transporter, glycolysis and gluconeogenesis genes from adult WT and KO mice (n=5 or 8 per genotype, respectively); B) Relative muscle mRNA expression of the indicated glucose transporter, glycolysis and gluconeogenesis genes from adult WT and KO mice (n=7 or 8 per genotype, respectively). Data were analyzed by Student's t-test or two-way ANOVA (in the case of GSIS). Values are expressed as mean +/- s.e.m. *P<0.05; **P<0.01; ***P<0.001. Adult mice were 9-12months of age. Source data are provided as a Source Data file.

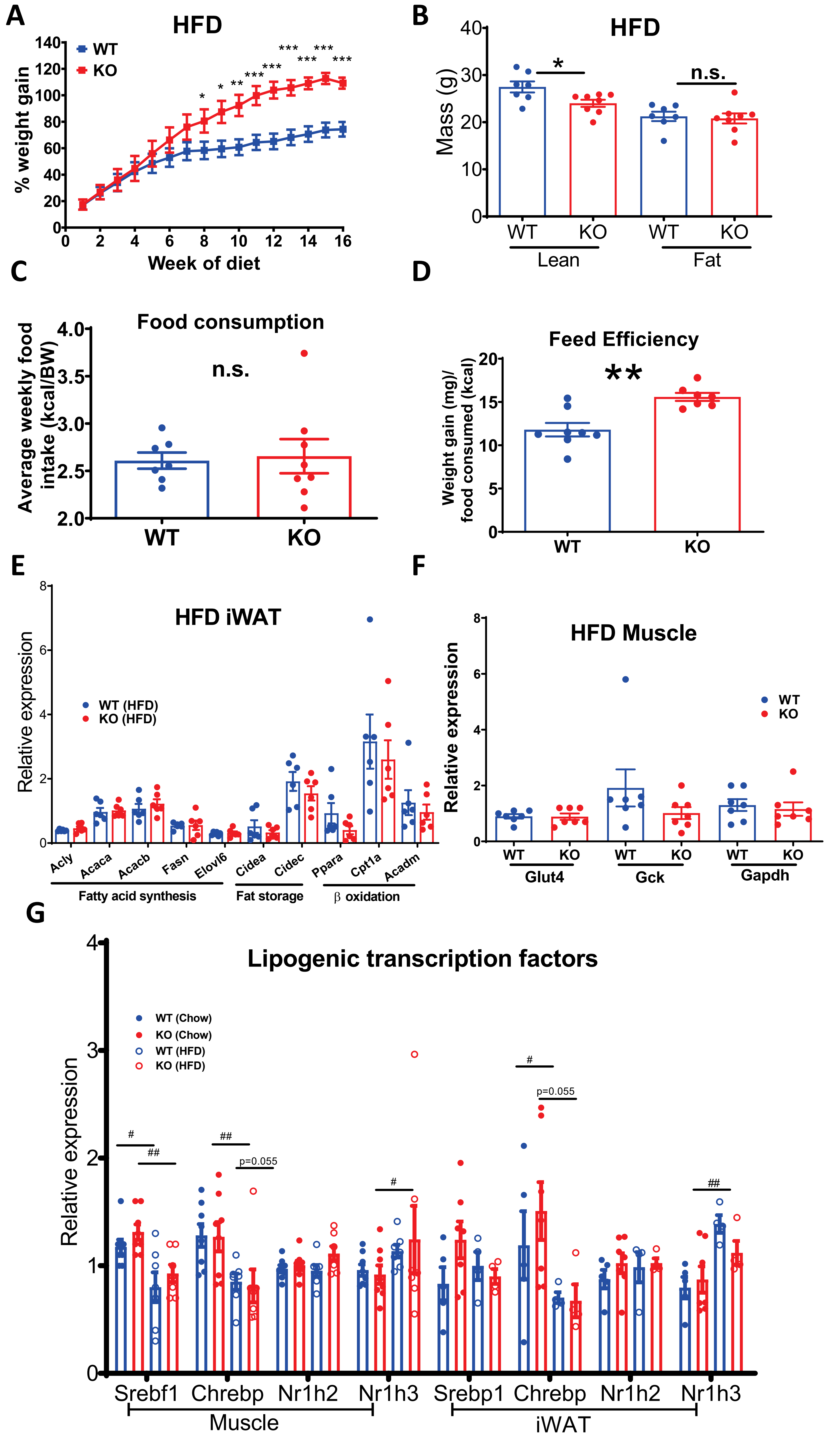
A
HFD
WT
KO
% weight gain
Week of diet
B
HFD
Mass (g)
*
n.s.
WT KO WT KO
Lean
Fat
C
Food consumption
n.s.
Average weekly food intake (kcal/BW)
WT
KO
D
Feed Efficiency
**
Weight gain (mg)/ food consumed (kcal)
WT
KO
E
HFD iWAT
WT (HFD)
KO (HFD)
Relative expression
Acly
Acaca
Acacb
Fasn
Elovl6
Cidea
Cidec
Ppara
Cpt1a
Acadm
Fatty acid synthesis
Fat storage
β oxidation
F
HFD Muscle
WT
KO
Relative expression
Glut4
Gck
Gapdh
G
Lipogenic transcription factors
WT (Chow)
KO (Chow)
WT (HFD)
KO (HFD)
Relative expression
p=0.055
Srebf1
Chrebp
Nr1h2
Nr1h3
Srebp1
Muscle
iWAT

Supplementary Fig 5. **Tmem127 deletion protects against high-fat diet (HFD)-induced hepatic steatosis and insulin resistance.** A) Weight gain relative to start body weight of WT and Tmem127 KO male mice on a 60% kcal fat diet for 16 weeks (n=8 per genotype, starting age 3-5 months); B) Relative lean and fat mass of WT and KO mice after 16 weeks of HFD (n=7 or 8 per genotype, respectively); C) Average weekly calorie intake per body weight of singly housed WT and KO mice on a HFD (n=8 or 7 per genotype, respectively); D) Feed efficiency calculated as the weight gain per kcal food intake, averaged per week) of singly housed WT and KO mice on a HFD (n=8 per genotype); E) Relative iWAT mRNA expression of the indicated fatty acid synthesis, storage and oxidation gene expression from HFD-fed male WT and KO mice (n=8 per genotype, 9-11 mo-old) measured by RT-PCR; F) Relative muscle mRNA expression of the indicated glucose transport and metabolism gene expression from HFD-fed male WT and KO mice (n=7 per genotype, 9-11 mo-old) measured by RT-PCR; G) Relative muscle and iWAT mRNA expression of the indicated lipogenic transcription factor genes from chow or HFD-fed male WT and KO mice (n=8 per genotype, 9-11 mo-old); data were analyzed by Student's t-test. Srebf1, Chrebp values are significantly different per diet, but not per genotype in both tissues. Other values are non-significant. Data were analyzed by Student's t-test and/or ANOVA. Values are expressed as mean +/- s.e.m. *P<0.05; **P<0.01; ***P<0.001; # comparisons between diet; * comparisons between genotype $^{\#}$ or *P<0.05; $^{\#\#}$ or **P<0.01; $^{\#\#\#}$or ***P<0.001. Source data are provided as a Source Data file.

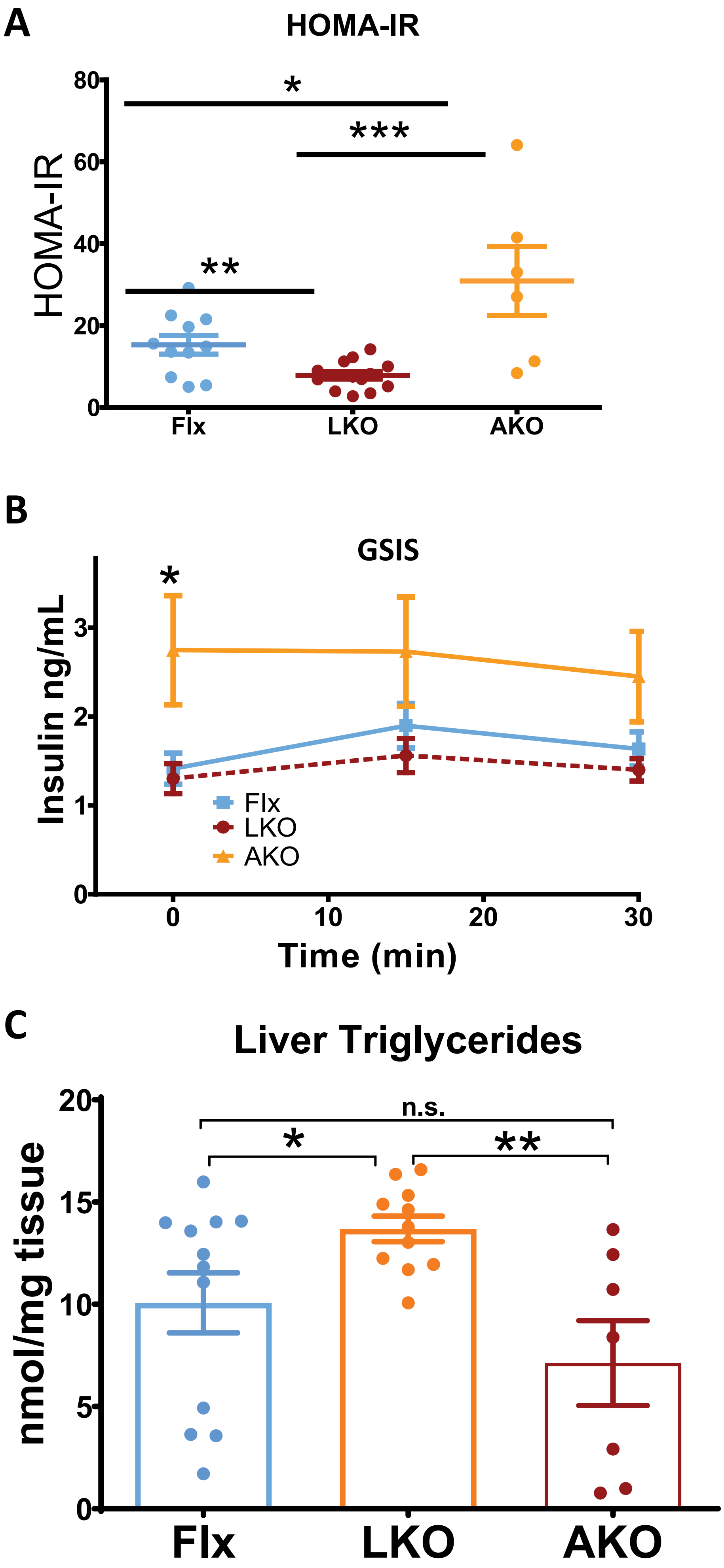

A
HOMA-IR
HOMA-IR
80
60
40
20
0
*
***
**
Flx
LKO
AKO
B
GSIS
Insulin ng/mL
*
3
2
1
0
Flx
LKO
AKO
0
10
20
30
Time (min)
C
Liver Triglycerides
nmol/mg tissue
20
15
10
5
0
n.s.
*
**
Flx
LKO
AKO

Supplementary Fig 6. **Liver-specific *Tmem127* deficient mice show improved glucose metabolism whereas adipose-specific Tmem127 deficient mice are insulin resistant**. A) Homeostatic model assessment -insulin resistance index (HOMA-IR) of adult male Flx, LKO and AKO mice (n= 11 Flx, 14 LKO, 6 AKO), data were analyzed by Student's t-test. Values are expressed as mean +/- s.e.m. *P<0.05; **P<0.01; ***P<0.001. B) Glucose-stimulated insulin secretion (GSIS) of adult Flx, LKO and AKO mice (n=9 Flx, n=6 LKO, n=5 AKO); e); C) Liver triglyceride content of fed Flx, LKO and AKO (n=12, 11 and 7, respectively). Data were analyzed by ANOVA; *p<0.05. Source data are provided as a Source Data file.

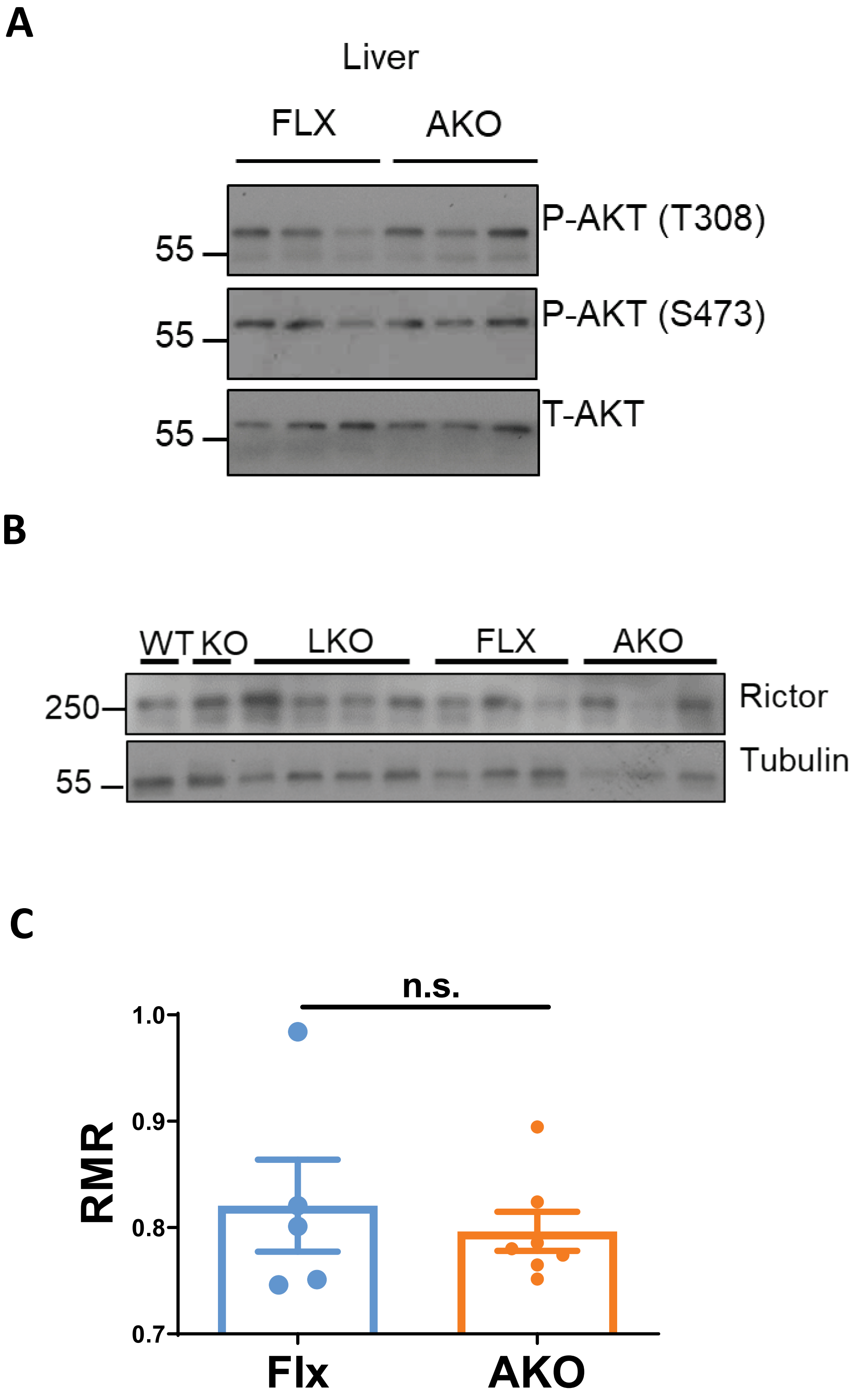
A
Liver
FLX
AKO
55
55
55
P-AKT (T308)
P-AKT (S473)
T-AKT
B
WT
KO
LKO
FLX
AKO
250
55
Rictor
Tubulin
C
n.s.
1.0
0.9
0.8
0.7
RMR
Flx
AKO

Supplementary Fig 7. Adipose-specific *Tmem127* deletion and hepatic insulin signaling. A) Western blot of liver lysates of Flx or AKO chow-fed, adult male (n=3 per genotype), probed with phosphorylated Akt at Ser473 or Thr308 or total Akt; B) Western blot of liver lysates of Flx (n=3); LKO (n=4); AKO (n=3), and one WT and one KO adult male, chow fed samples, probed with Rictor or Tubulin; C) Resting metabolic rate (RMR) calculated from the five lowest values of the adjusted oxygen consumption (VO2) in mL/hr/g lean body mass in 24h of Flx (n=5) and AKO (n=7) chow-fed, adult male mice. Data were analyzed by two-way ANOVA (n.s., non-significant). Source data are provided as a Source Data file.

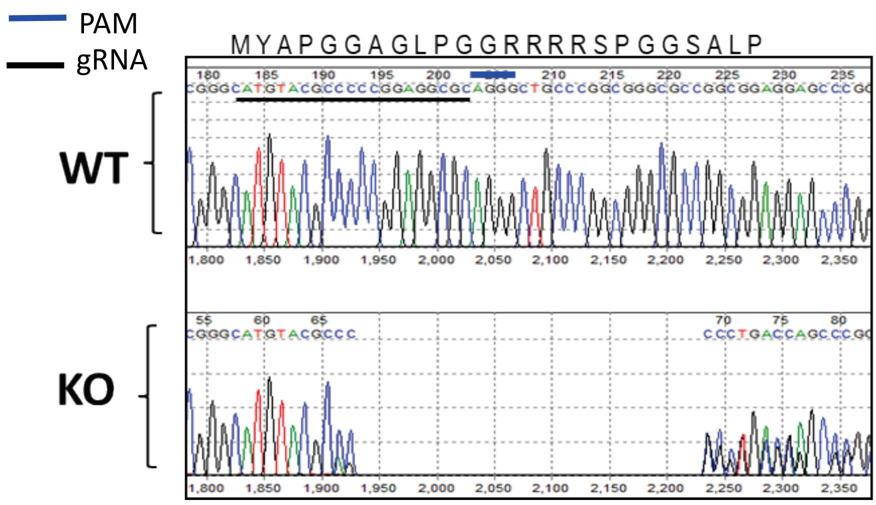
PAM
gRNA
M Y A P G G A G L P G G R R R R S P G G S A L P
WT
KO

Supplementary Fig 8. **Characterization of CRISPR-Cas9 mediate-*TMEM127* KO HepG2 cells.** Sanger sequencing of a HepG2 clone carrying a heterozygous deletion of exon 2 of the TMEM127 gene (KO). Guide RNA sequence is indicated with a black bar and PAM sequence is indicated with a blue bar.

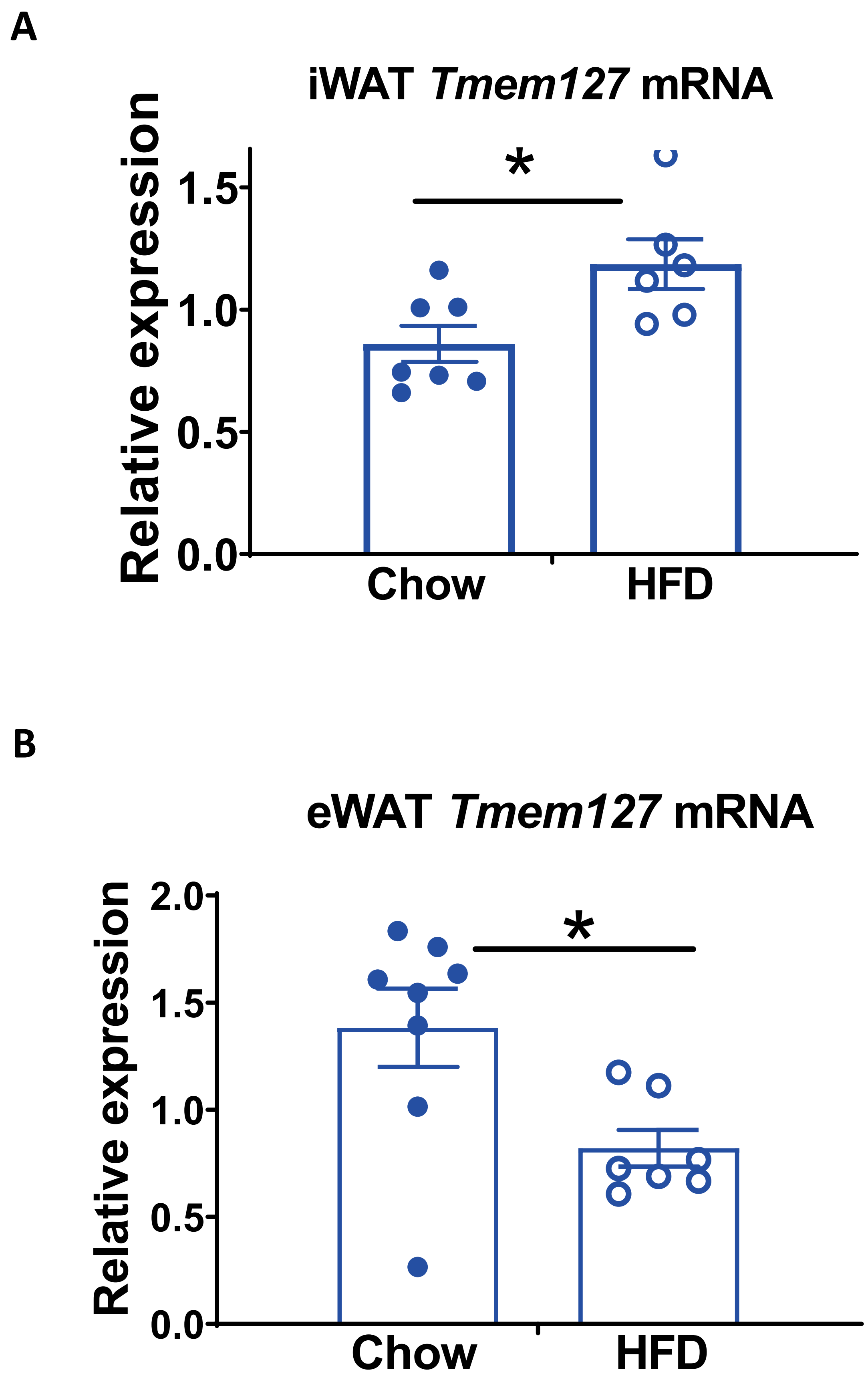
A
iWAT *Tmem127* mRNA
Relative expression
1.5
1.0
0.5
0.0
*
Chow
HFD
B
eWAT *Tmem127* mRNA
Relative expression
2.0
1.5
1.0
0.5
0.0
*
Chow
HFD

Supplementary Fig 9. ***Tmem127*** **mRNA expression of in fat tissues of wild-type adult male mice under chow or high-fat diet (HFD)**, quantified by real time PCR and displayed as relative expression normalized to the *Tfiib* gene: A) results from inguinal white adipose tissue (iWAT; n= chow n=7; HFD n=6; B) results from epididymal white adipose tissue (eWAT; chow n=8; HFD n=7). Data were analyzed by Student's t test; *p<0.05. Source data are provided as a Source Data file.

Supplementary Table 3- Liver TMEM127 mRNA levels and metabolic parameters in patients with fatty liver disease

Variable	Overall P	NAFLD+NASH P	NASH P
Age	0.052	0.058	0.201
BMI	0.019	0.038	0.041
AST	0.798	0.703	0.679
ALT	0.877	0.796	0.379
Insulin	0.005	0.018	0.036
Hba1C	0.019	0.004	0.544
Cholesterol	0.138	0.294	0.904
Triglyceride	0.015	0.075	0.307
INR	0.716	0.323	0.656
Glucose	0.096	0.277	0.934
Weight	0.838	0.693	0.894
HOMA-IR	0.011	0.050	0.203
NAFLD SCORE*	0.349	0.297	0.449

P values (Spearman's Correlation Coefficient) of TMEM127 expression: 'Overall P': all groups combined controls vs. nonalcoholic fatty liver disease (NAFLD) vs. nonalcoholic steatohepatitis (NASH); 'NAFLD + NASH P', combined NAFLD and NASH vs. control; 'NASH P', NASH only vs control; HOMA-IR= homeostatic model assessment of insulin resistance, BMI= body mass index; AST= aspartate amino transferase, ALT= alanine amino transferase; *As reported in Ref (29) Harrison et al. Gut 57, 1441-7 (2008)

Supplementary Table 4. Clinical, metabolic and molecular features of the cohort of patients with fatty acid liver disease

Disease Status	Fibrosis Stage	*TMEM127* read counts*	T2DM	Insulin (µIU/L)	HOMA-IR	BMI
Control	0	496.23	Yes	7.90	2.03	36.27
Control	0	147.16	No	9.40	2.20	35.42
Control	0	416.00	Yes	10.80	0.00	31.00
Control	0	491.94	No	15.80	4.41	31.01
Control	0	283.59	No			28.23
Control	0	343.43	No	1.20	0.29	26.38
Control	0	385.07	No	5.40	0.00	
NAFLD	0	490.08	No	69.70	16.69	26.07
NAFLD	0	426.05	No	10.70	3.09	36.77
NAFLD	0	489.00	No	14.70	3.70	24.69
NAFLD	0	530.73	No	19.10	4.53	32.61
NAFLD	0	495.23	No	7.00	1.69	29.99
NAFLD	0	426.00	No	17.40	3.87	33.12
NAFLD	0	359.48	No	11.40	2.42	32.38
NAFLD	0	400.56	No	44.40	10.63	25.60
NAFLD	0	467.38	No	7.60	1.71	26.31
NAFLD	0	444.05	No	18.20	4.45	27.80
NAFLD	0	160.92	No	13.10	3.49	29.66
NAFLD	0	471.48	No	9.50	2.56	29.02
NAFLD	0	280.86	No	14.00	3.28	30.27
NAFLD	0	438.38	No	7.40	1.74	28.41
NAFLD	0	456.75	No	6.30	1.28	30.38
NAFLD	0	438.77	No	23.70	5.79	31.75
NAFLD	0	407.13	No	16.60	3.89	28.17
NAFLD	0	394.36	Yes	21.70	7.34	30.02
NAFLD	0	490.61	No	9.60	2.20	
NAFLD	0	503.62	No	16.40	4.01	
NAFLD	0	438.35	No	9.50	2.58	
NASH	1	495.40	No	15.00	3.93	27.61
NASH	1	451.00	No	26.70	6.13	32.93
NASH	1	524.18	No	116.40	38.23	37.49
NASH	1	556.00	No	52.60	12.08	36.71
NASH	1	469.22	No	27.30	6.40	31.16
NASH	1	550.35	No	41.30	11.63	36.67
NASH	1	500.57	Yes	35.20	13.99	37.27
NASH	1	349.03	No	32.50	9.15	35.44
NASH	1	475.02	Yes	235.20	66.79	36.65
NASH	1	412.65	No	25.70	6.79	27.55
NASH	1	456.46	No	8.70	2.08	34.49
NASH	1	435.18	No	65.70	14.60	32.61
NASH	1	471.56	Yes	4.60	0.99	26.88
NASH	1	410.39	No	8.40	5.08	
NASH	1	500.28	Yes	26.10	15.79	
NASH	1	484.91	No			
NASH	2	510.76	No	17.60	3.69	32.61
NASH	2	494.87	No	13.10	3.11	25.62
NASH	2	500.71	Yes	41.00	10.43	27.53
NASH	2	498.30	Yes	28.90	7.99	
NASH	2	488.55	Yes	24.70	9.51	
NASH	3	437.54	Yes	22.60	13.23	31.35
NASH	3	540.56	No	39.20	10.55	31.47
NASH	3	554.93	Yes	12.70	3.54	37.56

NAFLD=nonalcoholic fatty liver disease, NASH= nonalcoholic steatohepatitis, T2DM= type 2 diabetes, HOMA-IR= homeostatic model assessment of insulin resistance, BMI= body mass index, *normalized read counts

Species	Primer Name	Primer sequence
mouse	Acly_F	GCCAGCGGGAGCACATC
mouse	Acly_R	CTTTGCAGGTGCCACTTCATC
mouse	Acaca_F	GCC TCT TCC TGA CAA ACG AG
mouse	Acaca_R	TGA CTG CCG AAA CAT CTC TG
mouse	Acacb_F	ACTCGCTTTGGAGGCAACAG
mouse	Acacb_R	CGCAGCGATGCCATTGTTG
mouse	Fasn_F	CAG CAG AGT CTA CAG CTA CCT
mouse	Fasn_R	ACC ACC AGA GAC CGT TAT GC
mouse	Scd1_F	CTGACCTGAAAGCCGAGAAG
mouse	Scd1_R	GCGTTGAGCACCAGAGTGTA
mouse	Elovl6_F	TGC TGC ATC CAG TTG AAG AC
mouse	Elovl6_R	TGC CAT GTT CAT CAC CTT GT
mouse	Cidea_F	GCCTGCAGGAACTTATCAGC
mouse	Cidea_R	TGCTTGCAGACTGGGACATA
mouse	Cidec_F	ATGGACTACGCCATGAAGTCT
mouse	Cidec_R	CGGTGCTAACACGACAGGG
mouse	Ppara_F	AACATCGAGTGTCGAATATGTGG
mouse	Ppara_R	CCGAATAGTTCGCCGAAAGAA
mouse	Cpt1a_F	AGGGCCGATCATGGTTAACA
mouse	Cpt1a_R	CGTGTTGGATGGTGTCTGTC
mouse	Acadm_F	AGGTTTCAAGATCGCAATGG
mouse	Acadm_R	ACCTTCATCGCCATTTCTGC
mouse	Cd36_F	ATTCTCATGCCAGTCGGAGA
mouse	Cd36_R	TGGGTTTTGCACATCAAAGA
mouse	Glut2_F	GGCTAATTTCAGGACTGGTT
mouse	Glut2_R	TTTCTTTGCCCTGACTTCCT
mouse	Gck_F	ATGGCTGTGGATACTACAAGGA
mouse	Gck_R	TTCAGGCCACGGTCCATCT
mouse	G6Pase_F	TGCTGCTCACTTTCCCCACCAG
mouse	G6Pase_R	TCTCCAAAGTCCACAGGAGGT
mouse	Pepck1_F (Pck1)	GACAGCCTGCCCCAGGCAGTGA
mouse	Pepck1_R (Pck1)	CTGGCCACATCTCGAGGGTCAG
mouse	Glut4_F	GTGACTGGAACACTGGTCCTA
mouse	Glut4_R	CCAGCCACGTTGCATTGTAG
mouse	Srebf1_F	GATCAAAGAGGAGCCAGTG
mouse	Srebf1_R	TAGATGGTGGCTGCTGAGTG
mouse	Chrebptotal_F	CACTCAGGGAATACACGCCTAC
mouse	Chrebptotal_R	ATCTTGGTCTTAGGGTCTTCAGG
mouse	Chrebpa_F	CGACACTCACCCACCTCTTC
mouse	Chrebpa_R	TTGTTCAGCCGGATCTTGTC
mouse	Chrebpb_F	TCTGCAGATCGCGTGGAG
mouse	Chrebpb_R	CTTGTCCCGGCATAGCAAC
mouse	Nr1h2_F	ATAGTGGGTCACGAAGCAGC
mouse	Nr1h2_R	AGGGCAACAGAGTCGGAGAC
mouse	Nr1h3_F	AGGAGTGTCGACTTCGCAAA
mouse	Nr1h3_R	CTCTTCTTGCCGCTTCAGTTT
mouse	Cebpa_F	CAAGAACAGCAACGAGTACCG
mouse	Cebpa_R	GTCACTGGTCAACTCCAGCAC
mouse	Tfiib_F	TGGAGATTTGTCCACCATGA
mouse	Tfiib_R	GAATTGCCAAACTCATCAAAACT
mouse	Pdk1_F	GGCGGCTTTGTGATTTGTAT
mouse	Pdk1_R	ACCTGAATCGGGGGATAAAC
mouse	Pfkp-F	AGGAGGGCAAAGGAGTGTTT
mouse	Pfkp-R	TTGGCAGAAATCTTGGTTCC
mouse	Pgd-F	AGACAGGCAGCCACTGAGTT
mouse	Pgd-R	AAGTTCTGGGTTTCGCTCAA
mouse	Gapdh_F	AGGTCGGTGTGAACGGATTTG
mouse	Gapdh_R	TGTAGACCATGTAGTTGAGGTCA
mouse	Tmem127_3F	TCGGTATGCCTTTGCTCACATCCT
mouse	Tmem127_4R	ACTTCTTATGCTGTTGCTGCTGGG
human	ACC1_R	AGTGGGTCACCCCATTGTT
human	ACC1_F	TTCTAACAGGAGCTGGAGCC
human	FASN_R	TCTCCGACTCTGGCAGCTT
human	FASN_F	GCTCCAGCCTCGCTCTC
human	SCD1_R	GCAGCCGAGCTTTGTAAGAG
human	SCD1_F	GTTCTACACCTGGCTTTGGG
human	ELOVL6_R	GTCTCTGACCCTTGCAGTCTT
human	ELOVL6_F	CCTGGTCACAAACTGACTGCT
human	SREBF1c_F	AAAAATCCGCCGCGCCT
human	SREBF1c_R	GCATGTCTTCGAAAGTGCAA
human	CHREBPb_F	AGCCCAGCCTCAAGGAGC
human	CHREBPb_R	CTGCTGGCACAGGTTAATGG
human	LXRB_F	CGCTACAACCACGAGACAGA
human	LXRB_R	GAACTCGAAGATGGGGTTGA
human	LXRA_F	GAAGAAACTGAAGCGGCAAGA
human	LXRA_R	ACTCGAAGCCGGTCAGAAAA
human	TBP_F	TATAATCCCAAGCGGTTTGCTGCG
human	TBP_R	AATTGTTGGTGGGTGAGCACAAGG
human	G6PASE_F (G6PC)	ACTGGCTCAACCTCGTCTTTA
human	G6PASE_R (G6PC)	CGGAAGTGTTGCTGTAGTAGTCA
human	TMEM127_F	TCGGTATGCCTTTGCTCACATCCT
human	TMEM127_R	ACTTCTTATGCTGTTGCTGCTGGG
