# Supplementary material for: The tumor suppressor TMEM127 regulates insulin sensitivity in a tissue-specific manner
Source: Nat Commun. 2019 Oct 17;10:4720. doi: 10.1038/s41467-019-12661-0 (PMC6797792; doi:10.1038/s41467-019-12661-0)
Supplement: Supplementary file 3 — Reporting Summary [file 41467_2019_12661_MOESM3_ESM.pdf]

nature research

Corresponding author(s): Patricia L. M. Dahia

Last updated by author(s): Jun 12, 2019

Reporting Summary

Nature Research wishes to improve the reproducibility of the work that we publish. This form provides structure for consistency and transparency in reporting. For further information on Nature Research policies, see Authors & Referees and the Editorial Policy Checklist .

Statistics

For all statistical analyses, confirm that the following items are present in the figure legend, table legend, main text, or Methods section.

n/a	Confirmed	
☐	☒	The exact sample size (*n*) for each experimental group/condition, given as a discrete number and unit of measurement
☐	☒	A statement on whether measurements were taken from distinct samples or whether the same sample was measured repeatedly
☐	☒	The statistical test(s) used AND whether they are one- or two-sided<br>*Only common tests should be described solely by name; describe more complex techniques in the Methods section.*
☐	☒	A description of all covariates tested
☐	☒	A description of any assumptions or corrections, such as tests of normality and adjustment for multiple comparisons
☐	☒	A full description of the statistical parameters including central tendency (e.g. means) or other basic estimates (e.g. regression coefficient) AND variation (e.g. standard deviation) or associated estimates of uncertainty (e.g. confidence intervals)
☒	☐	For null hypothesis testing, the test statistic (e.g. *F*, *t*, *r*) with confidence intervals, effect sizes, degrees of freedom and *P* value noted<br>*Give P values as exact values whenever suitable.*
☒	☐	For Bayesian analysis, information on the choice of priors and Markov chain Monte Carlo settings
☒	☐	For hierarchical and complex designs, identification of the appropriate level for tests and full reporting of outcomes
☐	☒	Estimates of effect sizes (e.g. Cohen's *d*, Pearson's *r*), indicating how they were calculated

Our web collection on statistics for biologists contains articles on many of the points above.

Software and code

Policy information about availability of computer code

Data collection	N/A
Data analysis	N/A

For manuscripts utilizing custom algorithms or software that are central to the research but not yet described in published literature, software must be made available to editors/reviewers. We strongly encourage code deposition in a community repository (e.g. GitHub). See the Nature Research guidelines for submitting code & software for further information.

Data

Policy information about availability of data

All manuscripts must include a data availability statement. This statement should provide the following information, where applicable:

- Accession codes, unique identifiers, or web links for publicly available datasets
- A list of figures that have associated raw data
- A description of any restrictions on data availability

All data, including RNAseq gene-specific (TMEM127) read counts raw and normalized values are available as source file underlying each of the figures and supplementary figures/tables.

Field-specific reporting

Please select the one below that is the best fit for your research. If you are not sure, read the appropriate sections before making your selection.

☒ Life sciences ☐ Behavioural & social sciences ☐ Ecological, evolutionary & environmental sciences

For a reference copy of the document with all sections, see nature.com/documents/nr-reporting-summary-flat.pdf

Life sciences study design

All studies must disclose on these points even when the disclosure is negative.

Sample size	Sample size varied from experiment to experiment. Our experiments with animal cohorts (diet stress, ITT, GTT, etc) were selected ~6-8 per group as those were anticipated to identify statistical differences based on our initial observation of ~15-20% body mass difference between genotypes; experiments in tissue-specific mice were limited by the number of available mice of the appropriate genotype (usually ~6, but >3/ per genotype/per experiment); in cell lines experiments were replicated three or four times per genotype, as detailed in the figure legends
Data exclusions	In some western blots (e.g. tissue-specific KO and flx controls) we also included additional controls (WT and CMV-KO) that were not included in the display or quantifications.
Replication	Experiments were performed as independent biological replicates (primary cells or cell lines). For animal experiments, whenever independent cohorts were used for the same experiments this is described; otherwise, cohort size is indicate in figure legend
Randomization	In all in vivo experiments we attempted to maintain a similar number of samples in each tested group; samples were matched for genotype, gender and age
Blinding	All calorimetry studies were performed blind by independent investigators

Reporting for specific materials, systems and methods

We require information from authors about some types of materials, experimental systems and methods used in many studies. Here, indicate whether each material, system or method listed is relevant to your study. If you are not sure if a list item applies to your research, read the appropriate section before selecting a response.

Materials & experimental systems

n/a	Involved in the study	
☐	☒	Antibodies
☐	☒	Eukaryotic cell lines
☒	☐	Palaeontology
☐	☒	Animals and other organisms
☐	☒	Human research participants
☒	☐	Clinical data

Methods

n/a	Involved in the study	
☒	☐	ChIP-seq
☒	☐	Flow cytometry
☒	☐	MRI-based neuroimaging

Antibodies

Antibodies used	from Cell Signaling Technologies: phospho-S6 ribosomal protein/S235/236 (#2211), total S6 ribosomal protein (5G10) (#2217), phospho-AKT/S473 (#9271), phospho-AKT/T308 (#13038), total AKT (#9272), phospho-4EBP1 (##2855), Rictor (#2114), total ACACA/ACC (#3676), FASN (#3180), mTOR antibody for western (#2983) and immunoprecipitation (#2972); alpha-tubulin was from Sigma (#T9026); TMEM127 polyclonal antibody was from Bethyl laboratories (#A303-450A).
Validation	All antibodies are commercially available and have been validated by vendor and/or other publications, including our own previous manuscripts using many of these antibodies (PMID: 30030286; PMID: 29547888; PMID: 24334765;PMID: 20154675)

Eukaryotic cell lines

Policy information about cell lines

Cell line source(s)	ATCC Hep G2 [HEPG2] ATCC® HB-8065™; primary hepatocyte cells were obtained from three cohorts of mice and were not propagated
Authentication	The HepG2 cells used had been authenticated by ATCC and were the only source of HepG2 cells in our laboratory
Mycoplasma contamination	All cells used were tested for Mycoplasma Contamination periodically (~every 2 months) using an in-house validated PCR method that spans multiple substrains of Mycolpasma. An internal species-specific control gene was included in each PCR, and a positive control (mycoplasma contaminated DNA) was included in every reaction
Commonly misidentified lines (See ICLAC register)	*Name any commonly misidentified cell lines used in the study and provide a rationale for their use.*

Animals and other organisms

Policy information about studies involving animals; ARRIVE guidelines recommended for reporting animal research

Laboratory animals	C57BL/6J background (Tg(CMV-cre)1Cgn; Jackson Laboratory; Albumin-Cre (Tg(Alb1-cre)1Dlr; Jackson Laboratory; Adiponectin-Cre (Tg(Adipoq-cre)1Evdr; Jackson Laboratory; Tmem127 Flx available through MTA agreement with UTHSCSA
Wild animals	*Provide details on animals observed in or captured in the field; report species, sex and age where possible. Describe how animals were caught and transported and what happened to captive animals after the study (if killed, explain why and describe method; if released, say where and when) OR state that the study did not involve wild animals.*
Field-collected samples	*For laboratory work with field-collected samples, describe all relevant parameters such as housing, maintenance, temperature, photoperiod and end-of-experiment protocol OR state that the study did not involve samples collected from the field.*
Ethics oversight	All protocols approved by institutional IACUC under protocol 20100053X; Animal Welfare Assurance Number A3345-01

Note that full information on the approval of the study protocol must also be provided in the manuscript.

Human research participants

Policy information about studies involving human research participants

Population characteristics	A total of fifty-two individuals (20 males, 53.4%), ages 20-64 were included in the study.
Recruitment	Patients with suspected diagnosis of liver disease
Ethics oversight	Samples were collected after informed consent was obtained, in accord with ethical standards though Institutional Review Board -approved protocols at the Brooke Army Medical Center (BAMC) and University of Texas Health Science Center at San Antonio (UTHSCSA)

Note that full information on the approval of the study protocol must also be provided in the manuscript.
